# Clinical implications with tolvaptan on monitored bioimpedance-defined fluid status in patients with cirrhotic ascites: an observational study

**DOI:** 10.1186/s12876-020-01205-2

**Published:** 2020-03-05

**Authors:** Shunsuke Shiba, Po-sung Chu, Nobuhiro Nakamoto, Karin Yamataka, Nobuhito Taniki, Keisuke Ojiro, Akihiro Yamaguchi, Rei Morikawa, Aya Yoshida, Akihiko Ikura, Hirotoshi Ebinuma, Hidetsugu Saito, Takanori Kanai

**Affiliations:** 1 Division of Gastroenterology and Hepatology, Department of Internal Medicine, Keio University School of Medicine, 35 Shinanomachi, Shinjuku-ku, Tokyo, 160-8582 Japan; 2 Department of Gastroenterology and Hepatology, Tokyo Dental College Ichikawa General Hospital, 5-11-13 Sugano, Ichikawa City, Chiba, 272-8513 Japan; 3 International University of Health and Welfare Mita Hospital, 1-4-3 Mita, Minato-ku, Tokyo, 180-8329 Japan; 4 Division of Pharmacotherapeutics, Keio University School of Pharmacy, 1-5-30 Shibakoen, Minato-ku, Tokyo, 105-8512 Japan

**Keywords:** Ascites, Impedance, Liver cirrhosis, Vasopressin antagonism

## Abstract

**Background:**

Prognostic value or clinical implications of fluid status monitoring in liver cirrhosis are not fully elucidated. Tolvaptan, an orally available, selective vasopressin V2-receptor antagonist approved for hyponatremia in the United States and European Union. It is also used for cirrhotic ascites at a relatively low dose (3.75 mg to 7.5 mg) in Japan, exerts its diuretic function by excreting electrolyte-free water. We hypothesized that bioimpedance-defined dynamic changes in fluid status allow prediction of response of V2 antagonism and survival in cirrhotic patients.

**Methods:**

In this prospective observational study, 30 patients with decompensated liver cirrhosis who were unresponsive to conventional diuretics were enrolled. Detailed serial changes of body composition that were assessed by using non-invasive bioimpedance analysis (BIA) devices, along with biochemical studies, were monitored at 5 time points.

**Results:**

Sixteen patients were classified as short-term responders (53%). Rapid and early decrease of BIA-defined intracellular water, as soon as 6 h after the first dose (ΔICW_BIA_%-6 h), significantly discriminated responders from non-responders (AUC = 0.97, *P* < 0.0001). ΔICW_BIA_%-6 h was highly correlated with the change of BIA-derived phase angle of trunk, e.g. reduced body reactance operated at 50 kHz after 24 h of the first dose of tolvaptan. Lower baseline blood urea nitrogen and lower serum aldosterone were predictive of a rapid and early decrease of ICW_BIA_. A rapid and early decrease of ICW_BIA_ in response to tolvaptan was also predictive of a better transplant-free survival.

**Conclusions:**

BIA-defined water compartment monitoring may help predict short-term efficacy and survival in decompensated cirrhotic patients treated with tolvaptan.

## Background

Liver cirrhosis, one of the leading health threat worldwide, yields larger years lived with disability (YLDs) globally than either hepatitis or hepatocellular carcinoma (HCC) [1]. Annually, 5 to 10% of patients with compensated cirrhosis develop ascites [2]. Hospitalization due to refractory ascites is one of the most frequent reasons for health-care cost in liver cirrhosis [3]. An unmet need for the treatment of refractory cirrhotic ascites still exists.

Although with possible reported limitations [4, 5], the non-invasiveness of bioimpedance analysis (BIA) has charmed many researchers to survey its possible application in healthy and diseased populations. BIA has been showed to be an adequate tool for evaluation of total body water (TBW) and extracellular water (ECW) in cirrhotic patients with ascites, [6, 7] and has been reported to assess body cell mass (BCM) after trans-jugular intrahepatic porto-systemic shunt (TIPS) in liver cirrhosis [8]. Newly developed ascites is a cardinal symptom of acute decompensation of cirrhosis and may occur in the setting of acute-on-chronic liver failure characterized by dysfunction in multiple organ systems, including renal and cardiovascular failure [9]. The pathophysiology of ascites due to liver cirrhosis is thought to be multifactorial. Malnutrition, systemic inflammation, and exaggerated activation of the renin-angiotensin-aldosterone system (RAAS) [10] play roles in the pathogenesis of cirrhotic ascites. These factors are also common pathophysiological features of fluid control in hemodialysis patients due to chronic renal failure. Body composition and fluid status monitoring assessed by non-invasive BIA, especially intracellular water (ICW), have been shown to be of prognostic value in acute decompensated heart failure, [11] acute kidney injury under continuous hemodiafiltration, [12] and patients with chronic renal failure under hemodialysis [13, 14]. Therefore, BIA has also been shown to be an applicable tool for assessment of ICW, even in patients with possible over-hydration or vigorous fluid status changes. However, in decompensated liver cirrhosis, the prognostic value and clinical implications of fluid status monitoring are not fully elucidated.

Tolvaptan, a selective vasopressin 2 (V2) receptor antagonist, has been indicated for treatment of hypervolemic or euvolemic hyponatremia in the US. Its efficacy and safety have been reported for patients with hyponatremia [15, 16]. Tolvaptan inhibits aquaporin (AQP)-2 channel expression in the collecting ducts and increases the excretion of electrolyte-free water by increasing hypoosmolar urine [17]. In 2013, tolvaptan was approved for use in cirrhotic ascites, whether hyponatremic or not, in Japan at low doses (3.75 mg to 7.5 mg per day) as an add-on (to conventional diuretics) approach [18]. Efficacy and safety have been reported in Japanese cirrhotic patients, including cases with Child-Pugh-Turcotte (CPT) grade C cirrhosis with this low-dose and add-on approach [19, 20]. In a randomized control trial, tolvaptan has been implicated to improve survival of cirrhotic patients with ascites and hyponatremia, especially in cases whose hyponatremia was resolved by tolvaptan [21]. In recent two observational studies, diuretic responders to tolvaptan showed survival benefit [22, 23]. However, there were still conflicting previous reports regarding diuretic efficacy and safety of V2 antagonism used for hyponatremia in cirrhotic patients [24, 25]. Results from pilot studies of heart failure implicate that V2 antagonism not only retracts ECW but also works to decrease ICW, [26] however, research on how electrolyte-free water excretion that is caused by V2 antagonism affects body composition and fluid status in cirrhotic patients is still sparse. Therefore, the accumulation of evidence for detailed monitoring of fluid status may help its adequate application in cirrhotic individuals.

In this observational study, we hypothesized that the use of add-on low-dose tolvaptan for cirrhotic ascites changes BIA-defined fluid volumes in different compartments, which may be a useful marker for the prediction of its short-term efficacy, and thus long-term survival in these patients.

## Methods

### Study subjects and study design

This study is a single arm, clinical practice-based observation study of a prospectively recruited cohort. The aim of this study is to observe how BIA-defined fluid status changes after add-on tolvaptan administration in patients with cirrhotic ascites. The definition of changes in fluid status is by longitudinal comparison the difference of BIA-defined fluid volumes with those at time 0 by expressing into percent of change (Δ%) in each study subject. It is note-worthy that the main purpose of this study is to find out the usefulness of fluid status monitoring after V2 antagonism in cirrhotic patients, rather than to prove the usefulness of a V2 antagonist itself, since a previous study [18] has proved this point. The recruitment of this study was conducted between April 2015 and March 2017, and 30 study subjects were enrolled. Subjects that survived without liver transplantation were followed at least for 1 year after the start of tolvaptan. All the enrolled patients were affected by persistent cirrhotic ascites despite treatment with conventional diuretics, including loop diuretics and aldosterone antagonists, which is coherent to the inclusion criteria raised by Sakaida et al. [18] In short, a cirrhotic patient with more than moderate amount of ascites assessed by physical examination and an image study whose body weight is not apparently decreased by a dose increase of conventional diuretics for at least 7 days is considered for inclusion. Therefore, the inclusion criteria are still considered reasonable even though they are not strictly coherent to the definition of “refractory” ascites defined by the International Club of Ascites [27]. Exclusion criteria include: (a) any patient suffering from hypernatremia (serum sodium levels exceeding 145 mmol/L); (b) chronic kidney disease grade G5 (eGFR< 15 mL/min/1.73m^2^); (c) polydipsia due to psychiatric disorders at baseline; (d) active gastrointestinal bleeding; (e) established bacterial infection including spontaneous bacterial peritonitis; (f) hepatic encephalopathy over West-Haven grade II; (g) unstable hymodynamic, renal, or respiratory state, which may suggest ongoing acute-on-chronic liver failure; (h) any patient who undergoes artificial porto-systemic or peritoneovenous shunting; (i) a recent large volume paracentesis. The inclusion flow is shown in Figure S1 (Additional file). The Model for End-stage Liver Disease (MELD) score and the combined score for MELD and serum sodium concentration (MELDNa score) were calculated as previously described [28]. Baseline plasma and urine osmolality was measured by freezing point depression methods. Clinical background of study subjects is shown in Table 1. Outcomes of the observation include: (1) BIA-defined fluid change in ICW or ECW, with a sub-analysis by whether a reduction of 1.5 kg or more in body weight on day eight, by which short-term response of tolvaptan [20] is defined, and (2) transplant-free survival. The included study subjects were observed with a median duration of 257.5 days (range 10–810 days).

**Table 1:** Pretreatment clinical characteristics including baseline body compositions of the study subjects

Variables	***N*** = 30
**Age, years**	66.5 [46–87]
**Sex, M/F**	17 (57%) /13 (43%)
Etiology of liver cirrhosis	
HCV/ Alcoholism /NASH /Others	11 (37%)/10 (33%) /4 (13%)/15 (17%)
Child-Pugh-Turcotte class, B/C	15/15
MELD score	13.5 [8–29]
MELDNa score	18.5 [8–32]
MAP, mmHg	82 [65–106]
Baseline diuretics	
Furosemide, mg/day	22.5 [0–160]
Spironolactone, mg/day	37.5 [0–100]
**Pre-treatment biochemical studies**	
Total bilirubin, mg/dL	2.0 [0.3–12.6]
PT-INR	1.23 [0.96–2.42]
Serum sodium, mmol/L	133.8 [122.3–142.8]
Albumin, g/dL	2.4 [1.6–3.9]
BUN, mg/dL	20.8 [5.9–47.1]
Serum creatinine, mg/dL	1.00 [0.48–2.49]
ALT, IU/L	25 [7–75]
GGTP, IU/L	70 [11–359]
AVP, pg/mL	1.8 [0.8–6.3]
Aldosterone, pg/mL	208 [18–1200]
Serum osmolality, mOsm/kg. H_2_O	285 [250–295]
Urine osmolality, mOsm/kg. H_2_O	448 [59–838]
**Body Compositions; baseline**	
BMI, kg/m^2^	23.2 [16.7–32.7]
Skeletal muscle index, kg/m^2^	9.1 [7.1–16.0]
TBW_BIA_-c ^a^, L/m^2^	20.4 [17.9–24.0]
ECW_BIA_-c ^a^, L/m^2^	8.3 [6.7–10.1]
ICW_BIA_-c ^a^, L/m^2^	12.0 [9.6–14.0]
ECW_BIA_/TBW_BIA_	0.40 [0.37–0.46]

Data are shown as median with the range within brackets, or numbers

*Abbreviations*: *M* Male, *F* Female, *HCV* Hepatitis virus C, *MELD* Model for end-stage liver disease, *MAP* Mean arterial blood pressure, *PT-INR* Prothrombin time- international ratio, *BUN* Blood urea nitrogen, *ALT* Alanine transaminase, *GGTP* γ- glutamyl transpeptidase, *AVP* Arginine vasopressin, *BMI* Body mass index, *TBW* Total body water, *BIA* Bioimpedance analysis, *ECW* Extracellular water, *ICW* Intracellular water

*, *P* < 0.05; **, *P* < 0.01; ***, *P* < 0.0001

^a^TBW_BIA_-c, ECW_BIA_-c, ICW_BIA_-c were normalized by body surface area (m^2^)

### Tolvaptan regimen and data sampling

All the study subjects were admitted and continued taking the same dose of conventional diuretics (furosemide, spironolactone, or both,) after the add-on of tolvaptan at a low dose. All admitted study subjects were on sodium-restricted diets (less than 102 mmol/L Na per day). Water restriction was not forced, and therefore, daily water intake was not monitored. Enrolled study subjects were advised about sodium restriction by a team of clinical dietitians at multiple time points including at admission, and they were monitored on their adherence to diet advices by the nursing team. Tolvaptan was initiated orally at a dose of 3.75 mg once per day, on the first day after breakfast. The dose may be increased to 7.5 mg once per day on the fourth day, if no adverse effects were observed. The doses from 3.75 to 7.5 mg are relatively low as compared to the doses (30 to 60 mg) approved for hyponatremia in the United States or in the European Union. Serial blood tests were performed to monitor a possible abrupt elevation of serum sodium, and the onset of hepatic or renal dysfunction. The induction protocol for tolvaptan was managed mainly according to the instructions of the latest clinical practice guidelines for liver cirrhosis in Japan [29]. Since tolvaptan has been reported that free water excretion was noticed after 24 h of the first dose [30], body weight, blood/urine sampling, and measurement of the body composition and fluid volumes (described later) were performed at five different time points: immediately before the start of tolvaptan (time point = 0 h); 6 h after the initial dose of tolvaptan (6 h); 1 day after the initial dose of tolvaptan (24 h; on day two), 3 days after the initial dose of tolvaptan (72 h; on day four); and 7 days after the initial dose of tolvaptan (168 h, on day eight). Please refer to Figure S2 for the schema of data sampling.

### Measurement of body composition and fluid volumes using bioimpedance analysis

Height and weight were obtained with an accuracy of 0.1 cm and 0.1 kg, respectively. The body mass index (BMI) was calculated as weight (kg)/height (m) squared. Bioimpedance analysis (BIA) -defined total body water (TBW_BIA_), extracellular water (ECW_BIA_), intracellular water (ICW_BIA_) and body cell mass were measured and analyzed by using a BIA device (InBody S10; InBody Japan, Tokyo, Japan). BIA was performed using the whole body 8-electrode approach with a multi-frequency impedance analyzer (1, 5, 50, 250, 500, and 1000 kHz) in the supine position, as instructed by the manufacturer. Resistance (R), reactance (Xc), and the phase angle (PA) were measured at each frequency. PA is the arc tangent of Xc/R and represents the phase difference between voltage and current [31]. The PA measured at 50 Hz was used in this analysis. Previous studies suggested that a decreased PA correlates with decreased ICW defined by BIA (ICW_BIA_) [12, 32]. BIA has been approved and indicated for body fluid and ECW monitoring in Japan. Information about body composition, ECW_BIA_, ICW_BIA_ and TBW_BIA_ were obtained at specific time points as was mentioned above and illustrated in Figure S2 (Additional file). Fluid volumes were compared after normalization by patient’s body surface area calculated by the Mosteller Method [33].

### Sample size estimation

Sample size calculation was based on the hypothesis that electrolyte-free water excretion into urine by tolvaptan results in a net movement of free water between ICW and extravascular ECW, and finally, the intravascular volume [26]. By application of the results from previous published studies: (1) tolvaptan may cause 270 ± 241 ml/day free water excretion [30]; (2) ECW/TBW ratio is about 0.399 ± 0.012 in cirrhosis patients [6]; (3) the standard deviation of ICW or ECW detected after tolvaptan in 6 cases is about 2.3 L [34]; (4) short-term efficacy of tolvaptan is around 62%, [20] with two-sided testing and a presumption of α = 0.05 and β = 0.2, a sample size of 7 in either responder or non-responder group, that is, a total participants of 20, was chosen to detect a between-group difference of compartment free water change after tolvaptan administration, which we considered clinically meaningful. The sample size was increased to 30 participants, to account for potential dropouts.

### Statistical analysis

Data were analyzed using JMP12 statistical software (SAS Institute, Inc. Cary, NC, USA) and are expressed as median with range or mean ± standard deviation (SD), as appropriate. Non-parametric Kruskal–Wallis tests were used to assess differences between groups. Categorical variables were analyzed using chi-square analysis. Spearman correlation was used for correlation analysis. The area under the receiver operating characteristic (AUROC) analysis was performed to confirm the usefulness of various parameters for predicting outcome and generating optimal cut-offs based on the Youden Index. The DeLong method [35] was used to compare the differences between receiver operating characteristic (ROC) curves. To investigate the independent determining factors to predict the rapid decrease of ICW_BIA_, logistic regression models adjusted for covariates were generated. Cumulative percentage of survival was determined by Kaplan–Meier analysis and the differences between groups were compared using a log-rank test. *P* < 0.05 was considered statistically significant.

## Results

### Baseline clinical characteristics and adverse events

Baseline patient characteristics are shown in Table 1. Thirty patients, 13 (43%) women and 17 (57%) men, with a median age of 66.5 years (range 46–87 years), were consecutively enrolled. Study participants consisted of 15 Child-Pugh-Turcotte (CPT) class B (50%) and 15 class C (50%) patients, with a median MELD score of 13.5 (range 8–29). No abrupt increase in serum sodium concentration was noticed during the observation period. Except for one (3.3%) patient who suffered from HCC rupture with subsequent pre-renal insufficiency and obviously increased serum total bilirubin and hypernatremia (145.2 mmol/L), serum creatinine concentrations and total bilirubin remained stable for most patients irrespective of their responses to add-on low-dose tolvaptan (Figure S3 in Additional file).

### Sub-analysis by short-term response to tolvaptan

**Fig. 1:**
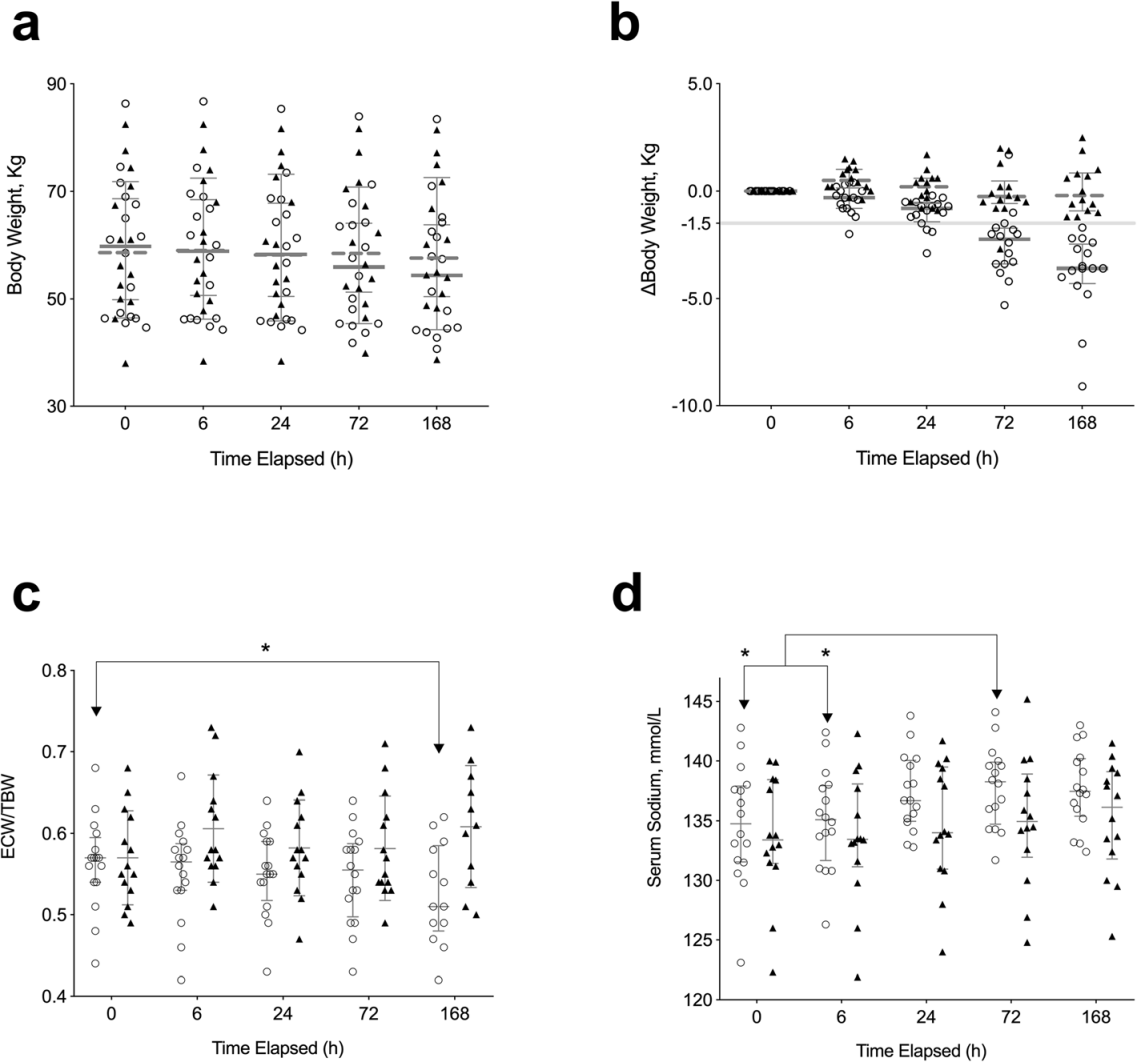
Serial monitoring of body weight, changes in BW, extracellular water to total body water ratio, and serum sodium concentration after add-on tolvaptan at low doses. Body weight (BW, in kilograms, **a**, changes in body weight (ΔBW, in kilograms, **b**, the extracellular water/total body water (ECW/TBW) ratio (using the bioimpedance analysis [BIA] method, **c**, and serum sodium concentration (in mmol/L, **d** were monitored as illustrated in Figure S2, at 0, 6, 24, 72, and 168 h (missing data: 6 for BIA at 168 h) after add-on tolvaptan at low doses was administered. Patients with 1.5 kg or more body weight reduction (horizontal light gray line in b) on day eight were defined as responders (open circles); all other patients were defined as non-responders (closed triangles). Medians with interquartile ranges are shown in bars. Abbreviations: ECW, extracellular water; TBW, total body water. *, *P* < 0.05

Sixteen patients (53%) with 1.5 kg or more body weight reduction on day eight were defined as responders to add-on tolvaptan at low doses, and the remaining 14 patients (47%) as non-responders (Fig. 1a-b). In responders, the ECW to TBW ratio (ECW/TBW) decreased gradually after the administration of tolvaptan and was significantly lower on day eight than on day 0 (Fig. 1c). This phenomenon was not observed in non-responders.

In univariate analysis (Table 2), age, sex, diuretics dosage, baseline serum sodium levels, urinary osmolality, and baseline body compositions evaluated by BIA, including the skeletal muscle index, and fluid volumes normalized by body surface areas, did not show any significant differences when comparing responders and non-responders. Responders had significantly lower levels of background total bilirubin (*P* = 0.034), alanine aminotransferase (ALT) (*P* = 0.004), AVP (*P* = 0.026), and aldosterone (*P* = 0.013) than non-responders. Responders also tended to be less azotemic (*P* = 0.085). Responders and non-responders did not differ in background presentation regarding HCC or portal hypertension (Table S1 in Additional file).

**Table 2:** Comparison of clinical characteristics in short-term responders and non-responders to tolvaptan

Variables	Responders	Non-responders	***P (uni)***
N	16 (53%)	14 (47%)	–
Age, years	71 [46–87]	65 [51–80]	0.227
Sex, M/F	8/8	9/5	0.484
ΔBW, at day 8, kg	−3.6 [−9.1 to −1.7]	−0.2 [−1.2 to + 2.5]	<.001***
Child-Pugh-Turcotte class, B/C	10/6	5/9	0.272
MELD score	13 [8–22]	16 [8–29]	0.164
MELDNa score	17 [8–30]	21 [12–32]	0.129
MAP, mmHg	81 [65–104]	82 [76–106]	0.533
Baseline diuretics			
Furosemide, mg/day	22.5 [0–60]	30 [0–160]	0.949
Spironolactone, mg/day	50 [25–100]	37.5 [0–75]	0.443
Pre-treatment biochemical studies			
Total bilirubin, mg/dL	1.5 [0.3–12.6]	3.5 [1.1–8.3]	0.034*
PT-INR	1.20 [0.96–1.75]	1.29 [1.03–2.42]	0.429
Serum sodium, mmol/L	134.8 [123.1–142.8]	133.4 [122.3–40.0]	0.692
Albumin, g/dL	2.5 [1.6–3.1]	2.4 [1.8–3.9]	0.917
BUN, mg/dL	18.3 [5.9–47.1]	23.6 [11.9–46.3]	0.085
Serum creatinine, mg/dL	1.00 [0.48–2.49]	1.00 [0.48–2.13]	0.819
ALT, IU/L	19.5 [7–45]	41 [11–75]	0.004**
GGTP, IU/L	59.5 [14–264]	92 [11–359]	0.262
AVP, pg/mL	1.35 [0.8–5.1]	2.3 [1.2–6.3]	0.026*
Aldosterone, pg/mL	113 [18–407]	316 [107–1200]	0.013*
Serum osmolality, mOsm/kg. H_2_O	286 [250–290]	282 [273–295]	0.880
Urine osmolality, mOsm/kg. H_2_O	448 [272–691]	452 [59–838]	0.350
**Body Compositions; baseline**			
BMI, kg/m^2^	23.1 [17.6–32.7]	23.7 [16.7–27.7]	0.574
Skeletal muscle index, kg/m^2^	9.1 [7.4–16.0]	9.3 [7.1–11.5]	0.603
TBW_BIA_-c ^a^, L/m^2^	20.2 [18.0–22.5]	20.8 [17.9–24.0]	0.575
ECW_BIA_-c ^a^, L/m^2^	8.3 [7.2–9.2]	8.3 [6.7–10.1]	0.755
ICW_BIA_-c ^a^, L/m^2^	12.0 [10.5–13.7]	12.4 [9.6–14.0]	0.418
ECW_BIA_/TBW_BIA_	0.40 [0.38–0.43]	0.40 [0.37–0.46]	0.418

Data are shown as median with the range within brackets, or numbers

*Abbreviations*: *M* Male, *F* Female, *HCV* Hepatitis virus C, *MELD* Model for end-stage liver disease, *MAP* Mean arterial pressure, *PT-INR* Prothrombin time- international ratio, *BUN* Blood urea nitrogen, *ALT* Alanine transaminase, *GGTP* γ- glutamyl transpeptidase, *AVP* Arginine vasopressin, *BMI* Body mass index, *TBW* Total body water, *BIA* Bioimpedance analysis, *ECW* Extracellular water, *ICW* Intracellular water

*, *P* < 0.05; **, *P* < 0.01; ***, *P* < 0.0001

^a^TBW_BIA_-c, ECW_BIA_-c, ICW_BIA_-c were normalized by body surface area (m^2^)

Four (13%) of our study subjects had baseline serum sodium concentrations below 130 mmol/L, the threshold defined for hyponatremia [36]. However, 17 (57%) subjects had serum sodium concentrations below 135 mmol/L, the threshold for increased mortality in patients listed for liver transplantation [28]. Serum sodium concentrations of responders to add-on, low-dose tolvaptan increased significantly at 72 h compared to those of non-responders (Fig. 1d), although the baseline serum sodium concentration did not differ significantly between responders and non-responders (Table 1).

### Temporal dynamics of compartmental fluid volumes evaluated by bioimpedance analysis

**Fig. 2:**
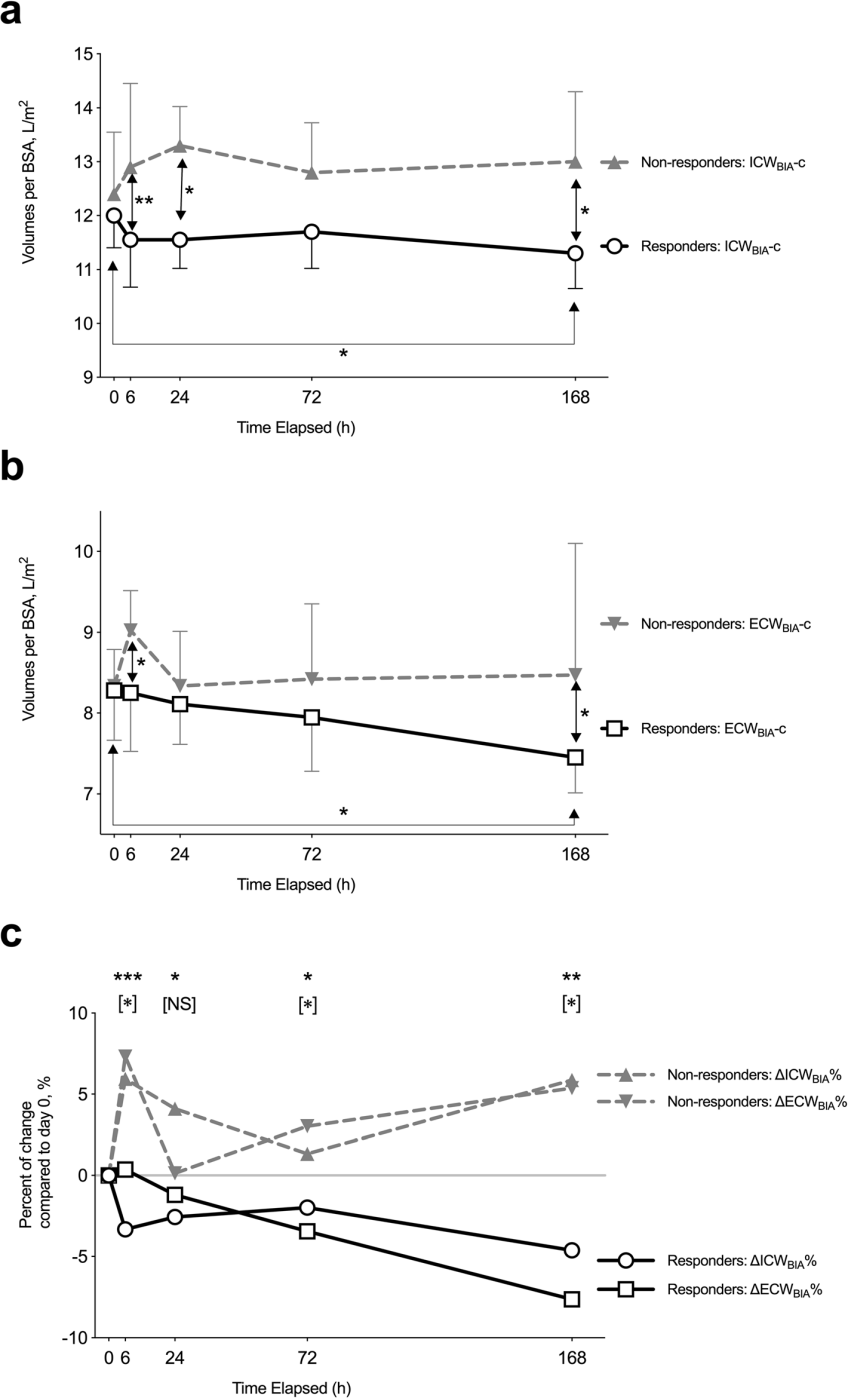
Serial monitoring of bioimpedance-defined intracellular water, extracellular water, and the percent of change in both parameters by bioimpedance analysis after add-on tolvaptan at low doses. Bioimpedance-defined intracellular body water standardized by body surface area (BSA) (ICW_BIA_-c, L/m^2^, **a**, extracellular body water standardized by BSA (ECW_BIA_-c, L/m^2^, **b**, and percent of change in both compartments compared to day 0 (panel **c**) were monitored as illustrated in Figure S2, at 0, 6, 24, 72, and 168 (missing data: 6 for BIA at 168 h) hours after add-on tolvaptan at low doses was administered. Patients with 1.5 kg or more body weight reduction on day eight were defined as responders (open circles for ICW_BIA_; open squares for ECW_BIA_; with thin lines), and others as non-responders (closed upward triangles for ICW_BIA_; closed downward triangles for ECW_BIA_; with dashed lines). Medians with interquartile ranges are shown in bars. In panel c, *P*-values compared ICWs with respect to response are shown, whereas *P*-values referring to ECW_BIA_, comparisons are shown in brackets. Abbreviations: ICW, intracellular water; ECW, extracellular water; BIA; bioimpedance analysis; BSA, body surface area; NS, not significant. *, *P* < 0.05; **, *P* < 0.01; ***, *P* < 0.0001

At 6 h after the first administration, a dramatic and rapid decrease in BIA-defined ICW_BIA_-c was noticed in responders, followed by a more gradual decrease in ECW_BIA_-c (Fig. 2a-b). ICW_BIA_-c was significantly lower in responders at 6 h, 24 h, and 168 h (Fig. 2a), and ECW_BIA_-c was significantly lower at 6 h and 168 h (Fig. 2b), than in non-responders. In responders, but not in non-responders, significant decreases in both ICW_BIA_-c and ECW_BIA_-c were observed (Fig. 2a-b).

**Fig. 3:**
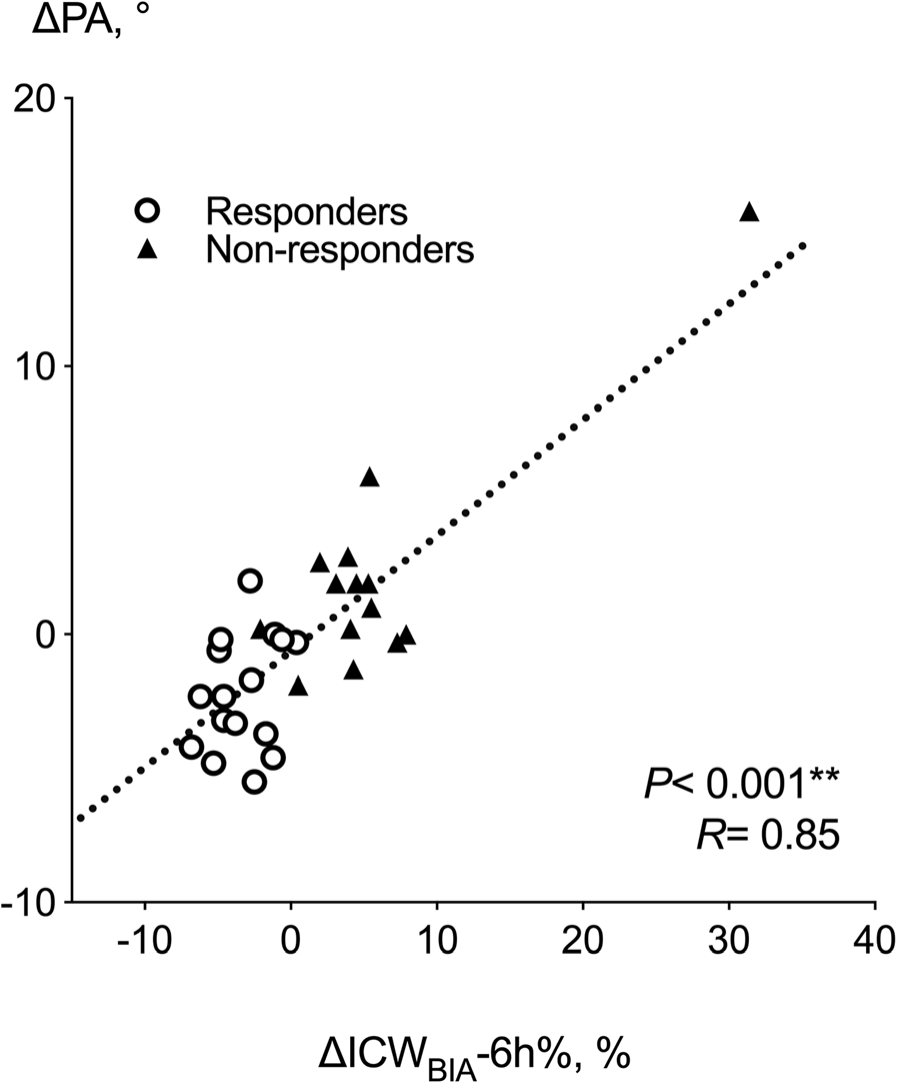
Correlation analysis of the change of the phase angle highly and the percent change of bioimpedance-defined intracellular water at 6 h after add-on tolvaptan. Patients with 1.5 kg or more body weight reduction on day eight were defined as responders (open circles), and others as non-responders (closed triangles). Phase angle (PA) was measured at 50 Hz. ICW, intracellular water; BIA, bioimpedance analysis; R, correlation coefficient. **, *P* < 0.001

The delay in the relative responses to ICW_BIA_ and ECW_BIA_ over time might imply a fluid volume shift from the ICW_BIA_ to the ECW_BIA_. The differences between responders and non-responders were most prominent and significant at 6 h after first administration of add-on tolvaptan (ΔICW_BIA_%-6 h). In responders, a gradual reduction in the change in ECW (ΔECW_BIA_%) was observed starting at around 24 h after administration of add-on tolvaptan, and continued thereafter (Fig. 2c). Change of the phase angle measured at 50 Hz by bioimpedance analysis was highly and significantly associated with ΔICW_BIA_%-6 h (Fig. 3). Detailed changes of resistance and reactance pre and post tolvaptan in individual cases are shown in Figure S4 (Additional file).

### Rapid and early decrease in ICW_BIA_ predicts short-term efficacy of add-on tolvaptan at low doses

**Fig. 4:**
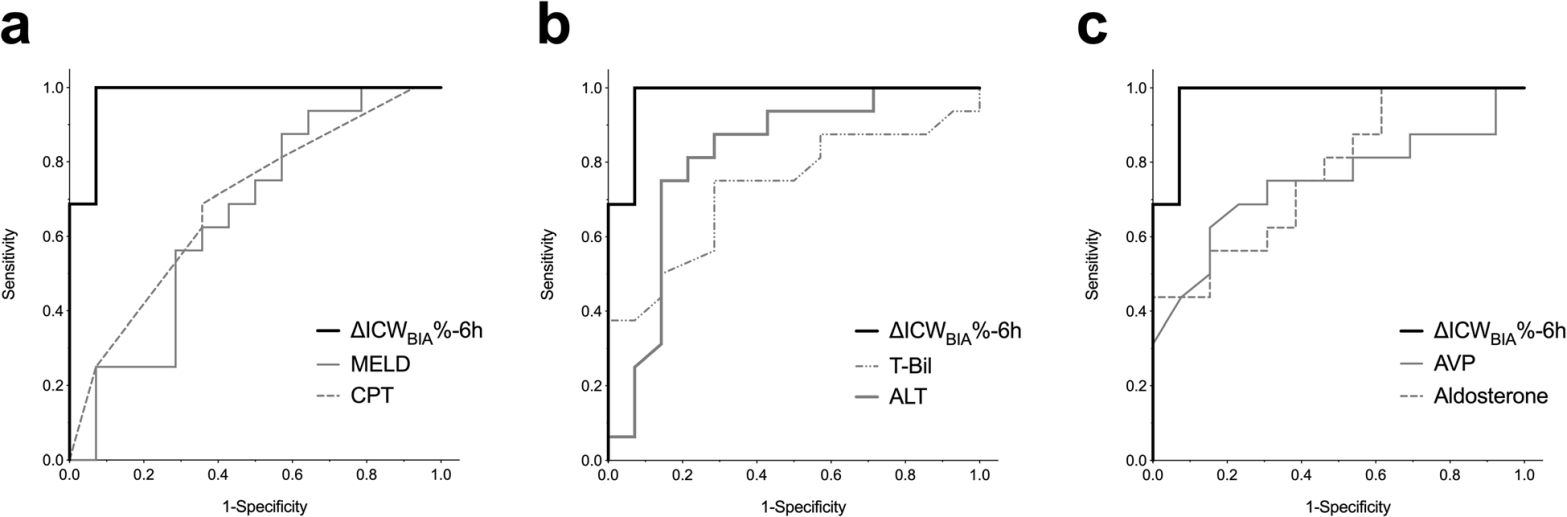
Receiver operating characteristic curves for predicting response to add-on tolvaptan. Comparison of the prognostic value of various parameters for the discrimination of responders and non-responders to add-on tolvaptan treatment is shown. The receiver operating characteristic (ROC) curves for the percent change in bioimpedance-defined intracellular water at 6 h after add-on tolvaptan (ΔICW_BIA_%-6 h), and other parameters are shown. Detailed statistics, including statistical comparison of the ROC curves by the DeLong method, are presented in Table S2. Abbreviations: MELD, model for end-stage liver disease; CPT, Child-Pugh-Turcotte; T-Bil, total bilirubin; AST, aspartate aminotransferase; ALT, alanine aminotransferase; AVP, arginine vasopressin

Rate of ICW_BIA_ volume change at 6 h after the first administration of add-on, low-dose tolvaptan (ΔICW_BIA_%-6 h), along with other factors that showed a significant or a tendency of significant difference between responders and non-responders (Table 1), were included in the ROC analyses to confirm their potential for response prediction (Fig. 4 and Table S2 in the Additional file). A ΔICW_BIA_%-6 h below 0, indicating a decreased ICW_BIA_ in response to tolvaptan compared to baseline, resulted to have the most significant diagnostic value (AUC = 0.98), as assessed using the DeLong method (Table S2 in the Additional file).

### Clinical parameters that predicted a rapid and early decrease of ICW_BIA_ in response to tolvaptan

In Table 3, clinical baseline parameters were compared between cases whose ICW_BIA_ decreased or increased after 6 h of the first dose of tolvaptan. Age, sex, baseline diuretics, serum sodium, creatinine, serum and urine osmolality did not differ significantly. Baseline MELDNa score, total bilirubin, ALT, serum aldosterone were significantly factors predicting a rapid and early decrease of ICW_BIA_ by univariate analysis. In multivariate analysis, serum aldosterone, along with blood urea nitrogen were found to be possible significant predictive factors for the rapid and early decrease of ICW_BIA_, after adjustment for MELDNa, total bilirubin, ALT, and/or AVP in various models (Table S3 in the Additional file).

**Table 3:** Comparison of clinical characteristics in cases whose ICW_BIA_ decreased or increased after six hours of add-on tolvaptan

Variables	ICW_**BIA**_ Decreased	ICW_**BIA**_ Increased	***P (uni)***
**N**	17 (57%)	13 (43%)	–
**Age, years**	69 [46–87]	65 [51–80]	0.325
**Sex, M/F**	9/8	8/5	0.721
**Background clinical profile**			
Child-Pugh-Turcotte B/C	11/6	4/9	0.139
MELD score	13 [8–23]	16 [8–29]	0.075
MELDNa score	17 [8–30]	21 [13–32]	0.047*
MAP, mmHg	81 [65–104]	82 [76–106]	0.818
Baseline diuretics			
Furosemide, mg/day	25 [0–160]	20 [0–80]	0.589
Spironolactone, mg/day	25 [0–100]	50 [0–75]	0.825
**Pre-treatment biochemical studies**			
Total bilirubin, mg/dL	1.5 [0.3–12.6]	3.5 [1.1–8.3]	0.040*
PT-INR	1.20 [0.96–1.75]	1.33 [1.03–2.42]	0.476
Serum sodium, mmol/L	135.6 [123.1–142.8]	133 [122.3–40.0]	0.368
Albumin, g/dL	2.6 [1.6–3.1]	2.3 [1.8–3.9]	0.867
BUN, mg/dL	18.2 [5.9–47.1]	25.3 [11.9–46.3]	0.054
Serum creatinine, mg/dL	0.99 [0.48–2.49]	1.01 [0.48–2.13]	0.786
ALT, IU/L	20 [7–75]	39 [11–69]	0.019*
GGTP, IU/L	64 [14–264]	95 [11–359]	0.315
AVP, pg/mL	1.4 [0.8–5.1]	2.3 [1.2–6.3]	0.076
Aldosterone, pg/mL	116 [18–407]	333 [107–1200]	0.012*
Serum osmolality, mOsm/kg. H_2_O	287 [250–295]	280 [273–293]	0.659
Urine osmolality, mOsm/kg. H_2_O	441 [272–691]	485 [59–838]	0.267

Data are shown as median with the range within brackets, or numbers

*Abbreviations*: *M* Male, *F* Female, *MELD* Model for end-stage liver disease, *Map* Mean arterial pressure, *PT-INR* Prothrombin time- international ratio, *BUN* Blood urea nitrogen, *ALT* Alanine transaminase, *GGTP* γ- glutamyl transpeptidase, *AVP* Arginine vasopressin

*, *P* < 0.05; **, *P* < 0.01

**Fig. 5:**
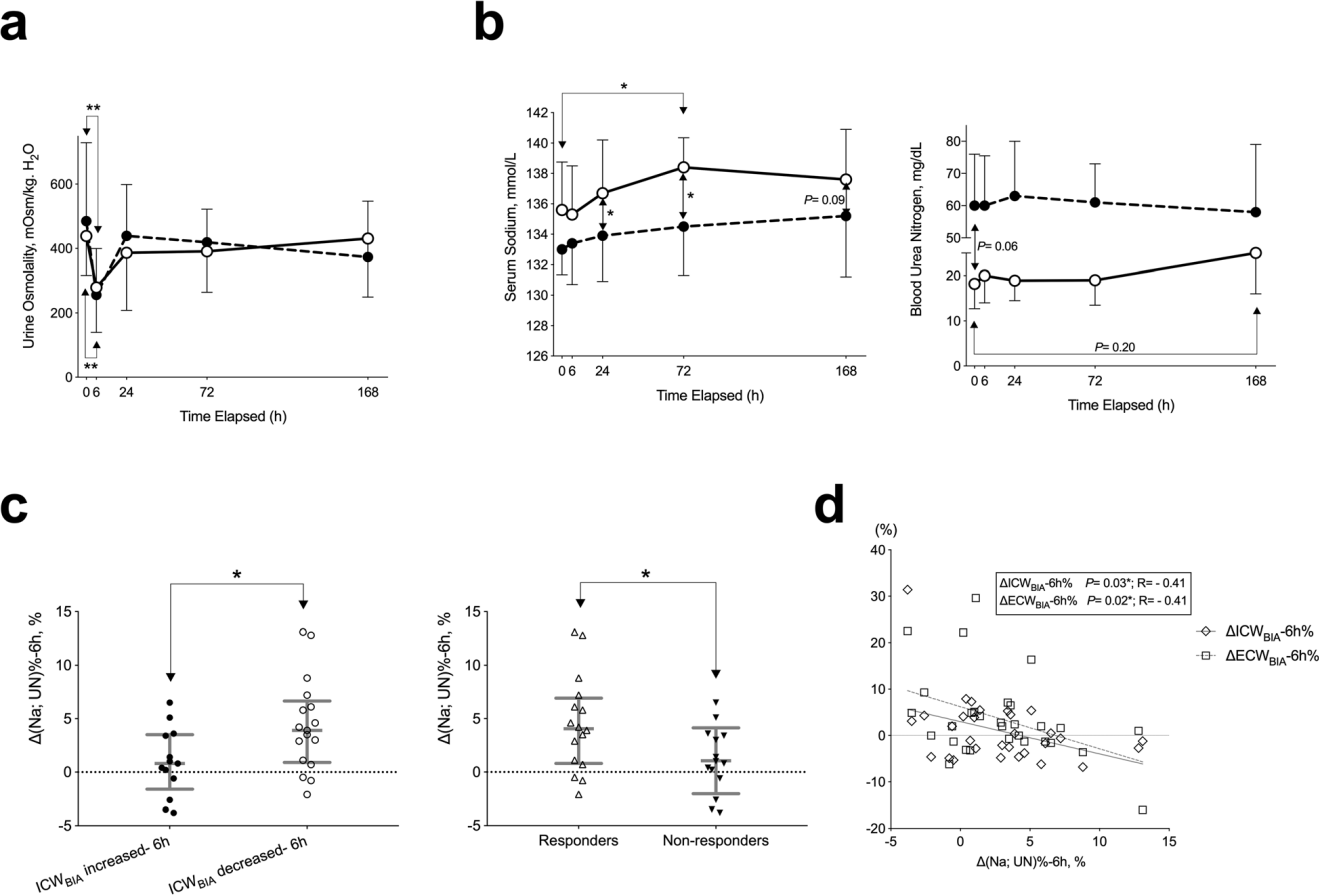
Serial monitoring of urine osmolality, serum sodium and blood urea nitrogen and the percent of change in both parameters in patients whose intracellular water decreased or increased after add-on tolvaptan. Patients whose bioimpedance-defined intracellular water (ICW_BIA_) decreased (open circles with lines) or increased (closed circles with dashed lines) after add-on tolvaptan were compared. Urine osmolality (mOsm/kg. H_2_O; a), serum sodium (mmol/L; b, left) and blood urea nitrogen (BUN, mg/dL; b, right) were monitored as illustrated in Figure S2, at 0, 6, 24, 72, and 168 h. The combined percent changes in both serum sodium (Na) and urea nitrogen (UN) were compared in ICW_BIA_ decreased/increased cases (c, left) and responders/non-responders (c, right). Spearman correlation analyses of the combined percent changes in both serum sodium (Na) and urea nitrogen (UN) and that of ICW_BIA_ and bioimpedance-defined extracellular water (ECW_BIA_) were shown in d. Medians with interquartile ranges are shown in bars. *, *P* < 0.05; **, *P* < 0.01

After the first dose of tolvaptan, a rapid decrease in urine osmolality was observed in both groups regardless of the change of ICW_BIA_ volumes at 6 h after the first dose of tolvaptan (Fig. 5a). However, in patients whose ICW_BIA_ decreased at 6 h, a significant increase in serum sodium was observed at 24 and 72 h, and the tendency still remained at 168 h. Additionally, blood urea nitrogen (BUN) levels showed a slower and gradual change; however, this tended to increase in patients whose ICW_BIA_ decreased at 6 h but stayed relatively stable in those whose ICW_BIA_ increased at 6 h (Fig. 5b). The increase in combined rate of change in serum sodium and BUN at 6 h, i.e., Δ (Na, UN)%-6 h, was significantly associated with decreased ICW_BIA_ at 6 h and the short-term response of add-on tolvaptan (Fig. 5c), as well as with changes in the rates of ICW_BIA_ and ECW_BIA_ at 6 h (Fig. 5d).

### Rapid and early decrease in ICW_BIA_ in response to tolvaptan significantly predicted a better clinical prognosis in decompensated cirrhosis

**Fig. 6:**
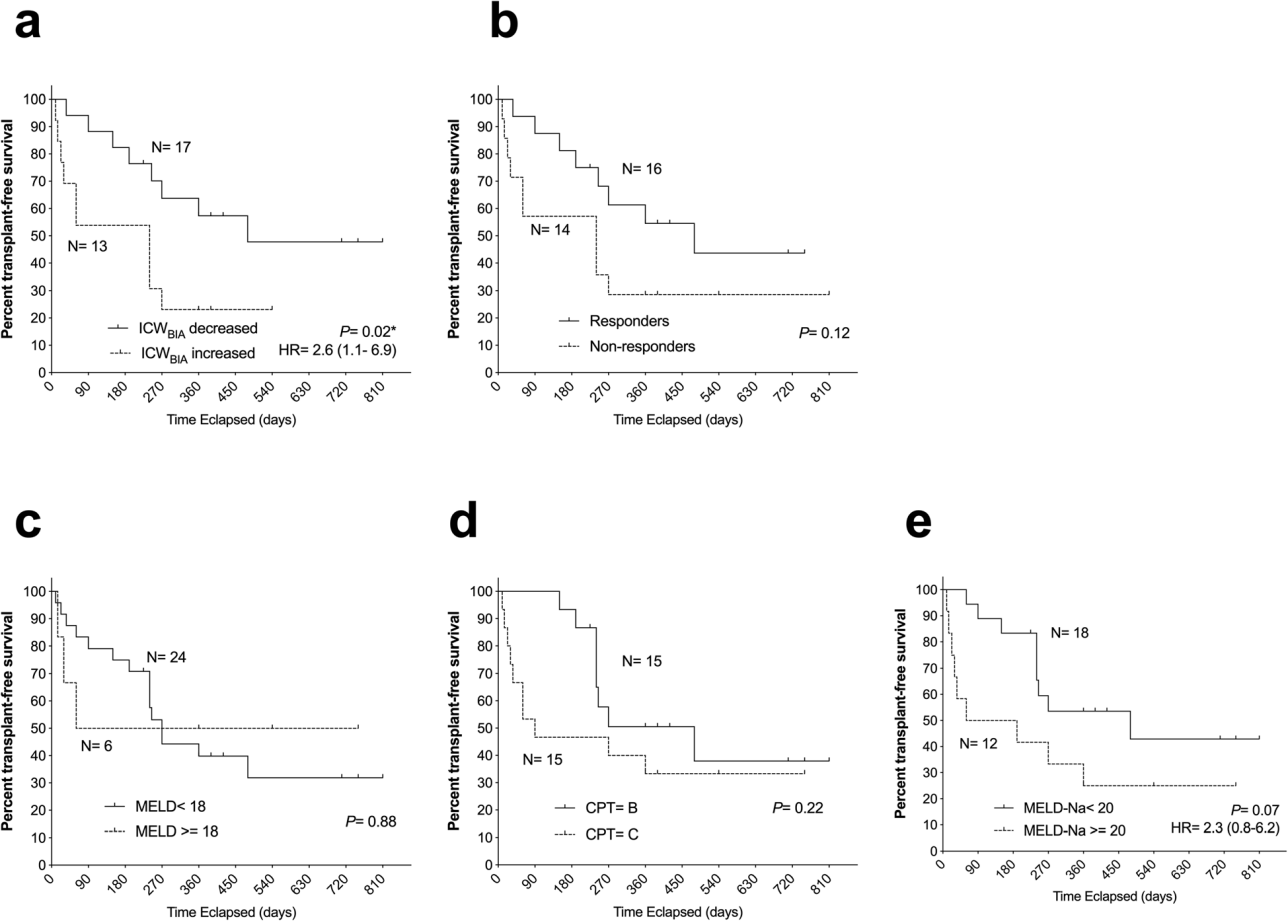
Kaplan–Meier analysis for long-term survival. In the 30 cases studied, transplant-free survival from the administration of add-on tolvaptan by Kaplan–Meier analysis is compared. Patients are stratified according to (**a**) bioimpedance-defined intracellular water (ICW_BIA_) decreases or increases after add-on tolvaptan; eight vs ten events; **b** response to tolvaptan; eight vs ten events; **c** Model for End-stage Liver Disease (MELD) score at 18; fifteen vs three events; **d** Child-Pugh-Turcotte (CPT) grade B or C; eight vs ten events; **e** MELDNa score at 20; nine vs nine events. *P-*values and hazard ratio from log-rank tests are shown. *P < 0.05

During the observation period, four patients underwent liver transplantation, and 14 patients died due to end-stage liver disease. The transplant-free survival is 40%. As shown in Fig. 6, although the response to add-on tolvaptan showed a tendency of longer survival in this study cohort (*P* = 0.12, Fig. 6a), the rapid and early decrease in ICW_BIA_ at 6 h after the first dose of tolvaptan significantly differentiated two groups of patients in terms of long-term survival (*P* = 0.02, Fig. 6b). Neither baseline MELD score nor Child-Pugh grade could differentiate the survival of patients beyond 90 days of observation (Fig. 6c-d); instead, the baseline MELDNa score tended to predict survival (*P* = 0.07, Fig. 6e). Despite having excluded five cases with advanced HCC (Barcelona Clinic Liver Cancer stage C or D) from the analysis, the rapid ICW_BIA_ decrease in response to tolvaptan still significantly correlated with better long-term survival (*P* = 0.048; Figure S5 in the Additional file).

## Discussion

In the current study, we demonstrated a rapid and early decrease in phase angle (reactance), e.g. reduced ICW assessed by BIA after adding low dose tolvaptan, correlated significantly with short-term efficacy of amelioration of body fluid retention and the long-term survival. ΔICW_BIA_%-6 h predicted short-term efficacy of add-on tolvaptan at an accuracy of 97% and long-term survival in our study cohort. To our knowledge, this is the first real world clinical practice-based study to show the usefulness of BIA-assessed ICW change in response to electrolyte-free water excretion caused by V2 antagonism for both short-term efficacy and long-term survival, in patients of decompensated cirrhosis.

Kogiso et al. reported that ECW/TBW ratio significantly decreased in eight long-term responders to tolvaptan [19]. We also observed the same tendency in responders in this current study (Fig. 1c). BIA is also a useful tool to demonstrate that the loss of body weight in responders is mainly due to decreased TBW, rather than reduced body cell mass. Selberg et al. showed that higher BIA-derived phase angle (assumed to be determined by tissue cellularity, tissue hydration, and membrane potential) in 305 patients of liver cirrhosis predicted poorer survival [37]. This is coherent to our finding that a decreased phase angle after add-on tolvaptan (decreased ICW_BIA_) predicted better prognosis (Fig. 3).

Masuda et al. reported that tolvaptan reduced both ICW and ECW, evaluated by BIA in six patients with chronic kidney disease [34]. Masuda et al. also presumed that a rise in serum osmolality and resultant fluid shift from intracellular to interstitial and intravascular spaces would occur immediately after the administration of tolvaptan. With BIA, Nagayama et al. demonstrated different effects on fluid distribution between tolvaptan and furosemide in a patient with liver cirrhosis and chronic kidney disease [38]. Nomoto et al. showed that in patients with acute decompensated heart failure, diuresis due to tolvaptan caused no significant change of ECW/ICW ratio by using BIA, while that due to conventional diuretics decreased ECW/ICW ratio [11]. This finding suggested a net reduction of ICW might characterize tolvaptan from conventional diuretics. In the current study, we showed that a decrease in ICW_BIA_ occurred as soon as 6 h after the first dose of tolvaptan (Fig. 2), and its degree correlated with the increase in BUN and serum sodium (Fig. 5), which are the major determinant factors for both serum osmolality and urine-concentrating mechanisms in the renal medullary loops of Henle [39]. The increase in serum sodium and BUN (Fig. 4b) might help to induce a shift of free water from the intracellular compartment, which is less prominent in non-responders. Higher baseline BUN has also been reported as a negative predictive factor for the efficacy of tolvaptan for cirrhotic ascites [40, 41]. In this present study, the baseline BUN tented to be higher (*P* = 0.06, Table 1), and the follow-up BUN levels remained high in the non-responders (Fig. 5b).

The reason for why early extractable ICW_BIA_ after V2 antagonism unexpectedly correlates with survival (Fig. 5) is not easy to explain. Hiramine Y et al. demonstrated a positive effect of add-on tolvaptan on the prognosis of patients with cirrhotic ascites by analyzing 628 patients retrospectively, compared to conventional diuretics alone [42]. Higher baseline BUN levels, that are supposedly a result of the chronic vascular under-filling state due to cirrhosis, might also be a result of chronic diuretic use for ascites. Sone et al. showed that the chronic administration of furosemide, a loop diuretic agent, greatly reduced renal medullary contents of organic osmolytes in a murine model [43]. Conversely, a selective V2 antagonist did not produce a sufficient decrease in the content of organic osmolytes; while, an increase in taurine levels, an effective organic osmolyte, was observed following exposure to a selective V2 antagonist in a murine model [44]. Therefore, a well-preserved urine-concentrating ability that implicates preserved renal medullary osmolyte levels, favors a transient and immediate rise in osmolality after V2 antagonism, and is also assumed to be required for the immediate extraction of ICW_BIA_, a positive predictive parameter for tolvaptan efficacy in this study. Moreover, more severe hyponatremia is associated with an increase in ICW_BIA_ after add-on tolvaptan in our study cohort (Fig. 5b, left). This was coherent with a previous study showing that maintained serum sodium over 140 mmol/L is a significant predictor of response to tolvaptan [22]. Hyponatremia in cirrhosis is associated with more intractable ascites and greater impairments of renal function [36]. A higher degree of extractable ICW_BIA_ with low-dose of tolvaptan treatment might also suggest better-preserved renal function, and therefore, better survival potential.

Base on previous studies until early 2000s, some experts reported that cautions might be needed for the explanation of cross-sectional quantification of ICW or ECW by using BIA, especially in patients with altered body compositions such as kidney, heart, or liver diseases [4, 45]. In this current study, we used the longitudinal comparison in each individual for assessment, in order to lessen confounding effects such as possible anthropometric factors. In addition, in order to overcome the limitation of the direct use of BIA-defined volumes in diseased state, the use of BIA-derived “phase angle” to define changes in body compositions in cirrhosis has been focused [37, 46]. We also showed a significant and high correlation between the change of phase angle and ICW_BIA_ in a cross-sectional analysis (Fig. 3).

Still, there are other major limitations to this study. First, even though add-on low dose tolvaptan for cirrhotic ascites is generally approved and used in daily practice in Japan, it is still not a standard treatment internationally. This point might limit applicability of the results yielded by this study. In addition, although we monitored body composition through serial measurement of BIA within 1 week after administration of low-dose tolvaptan, the small sample size without a proper control group might limit the generalizability of this observation and made it difficult to control for effects of possible confounders with regard to long-term survival, which might cause selection bias. Further investigations are still warranted to determine whether and how low dose tolvaptan improves survival in patients with cirrhosis in a prospective setting. Moreover, although BIA for assessment of body composition is generally considered to be reproducible and accurate with few cost and invasiveness concerns [5], and the application of phase angle might meet some needs in populations with altered body composition, it is still not fully validated in many edematous states. This is possibly why BIA is not routinely applied in practice until now. Finally, since the concomitant use of conventional diuretics was maintained during the present study, the effect of monotherapy with tolvaptan to change fluid volumes in different compartments was not examined.

## Conclusions

With the serial monitoring of fluid status by BIA, we found that the rapid and early decrease in ICW_BIA_ predicted short-term efficacy of add-on tolvaptan at low doses. The ICW_BIA_ decrease in response to add-on tolvaptan was also predictive of survival in patients with decompensated cirrhosis. Even though there are still many technical limitations remaining, we believe that based on the evidence provided by this study, BIA-defined water compartment monitoring might play a role in the management and care for decompensated cirrhotic patients with ascites in the future.

## Supplementary information

**Additional file 1 Figure S1** Inclusion flow of the study subjects. **Figure S2** Study schema for tolvaptan administration and the serial monitoring of body compartments and biochemical studies. **Figure S3** Serial monitoring of estimated glomerular filtration rate and total bilirubin of the study subjects after add-on tolvaptan at low doses. **Figure S4** The resistance-reactance path graphs for responders (panel A) and non-responders (panel B) pre and post add-on tolvaptan at frequency of 50 kHz. **Figure S5** Kaplan–Meier analysis for long-term survival as stratified by bioimpedance-defined intracellular water response to add-on tolvaptan in cases without advanced hepatocellular carcinoma. **Table S1** Pretreatment clinical characteristics regarding hepatocellular carcinoma and portal hypertension of the study subjects **Table S2** Comparison between areas under the receiver operating characteristic curve of clinical parameters for differentiation between responders and non-responders. **Table S3** Models of multivariate analysis for predicting the rapid decrease of ICW_BIA._

## Data Availability

All datasets generated during the current study are available from the corresponding authors on reasonable request, and the results from all data analyzed during this study are included in the published article.

## Abbreviations

ALT: Alanine aminotransferase
AQP: Aquaporin
AVP: Arginine vasopressin
BIA: Bioimpedance analysis
CPT: Child-Pugh-Turcotte
ECW: Extracellular water
HCC: Hepatocellular carcinoma
ICW: Intracellular water
ICW_BIA_: Bioimpedance analysis-defined intracellular water
MELD: Model for End-stage Liver Disease
TBW: Total body water
YLDs: Years lived with disability
