# Supplementary material for: Clinical implications with tolvaptan on monitored bioimpedance-defined fluid status in patients with cirrhotic ascites: an observational study
Source: BMC Gastroenterol. 2020 Mar 5;20:53. doi: 10.1186/s12876-020-01205-2 (PMC7059268; doi:10.1186/s12876-020-01205-2)
Supplement: Supplementary file 1 — Additional file 1 Figure S1 Inclusion flow of the study subjects. Figure S2 Study schema for tolvaptan administration and the serial monitoring of body compartments and biochemical studies. Figure S3 Serial monitoring of estimated glomerular filtration rate and total bilirubin of the study subjects after add-on tolvaptan at low doses. Figure S4 The resistance-reactance path graphs for responders (panel A) and non-responders (panel B) pre and post add-on tolvaptan at frequency of 50 kHz. Figure S5 Kaplan–Meier analysis for long-term survival as stratified by bioimpedance-defined intracellular water response to add-on tolvaptan in cases without advanced hepatocellular carcinoma. Table S1 Pretreatment clinical characteristics regarding hepatocellular carcinoma and portal hypertension of the study subjects Table S2 Comparison between areas under the receiver operating characteristic curve of clinical parameters for differentiation between responders and non-responders. Table S3 Models of multivariate analysis for predicting the rapid decrease of ICWBIA. [file 12876_2020_1205_MOESM1_ESM.docx]

**Additional files**

**Clinical implications with tolvaptan on monitored bioimpedance-defined fluid status in patients with cirrhotic ascites: an observational study**

Shunsuke Shiba^1^, Po-sung Chu^1*^, Nobuhiro Nakamoto^1^, Karin Yamataka^1^, Nobuhito Taniki^1^, Keisuke Ojiro^1, 2^, Akihiro Yamaguchi^1^, Rei Morikawa^1^, Aya Yoshia^1^, Akihiko Ikura^1^, Hirotoshi Ebinuma^1, 3^, Hidetsugu Saito^1, 4^, and Takanori Kanai^1*^

^1^Division of Gastroenterology and Hepatology, Department of Internal Medicine, Keio University School of Medicine, 35 Shinanomachi, Shinjuku-ku, Tokyo 160-8582, Japan

^2^Department of Gastroenterology and Hepatology, Tokyo Dental College Ichikawa General Hospital, 5-11-13 Sugano, Ichikawa City, Chiba 272-8513, Japan

^3^International University of Health and Welfare Mita Hospital, 1-4-3 Mita, Minato-ku, Tokyo 180-8329, Japan

^4^Division of Pharmacotherapeutics, Keio University School of Pharmacy, 1-5-30 Shibakoen, Minato-ku, Tokyo 105-8512, Japan

*Corresponding authors: Po-sung Chu and Takanori Kanai, Division of Gastroenterology and Hepatology, Department of Internal Medicine, Keio University School of Medicine, 35 Shinanomachi, Shinjuku-ku, Tokyo 160-8582, Japan. Tel: +81-3-3353-1211; Fax: +81-3-3353-6247; E-mail: [pschu](mailto:chohakusyo@yahoo.co.jp)0928@iCloud.com and [takagast@z2.keio.jp](mailto:takagast@z2.keio.jp)

**
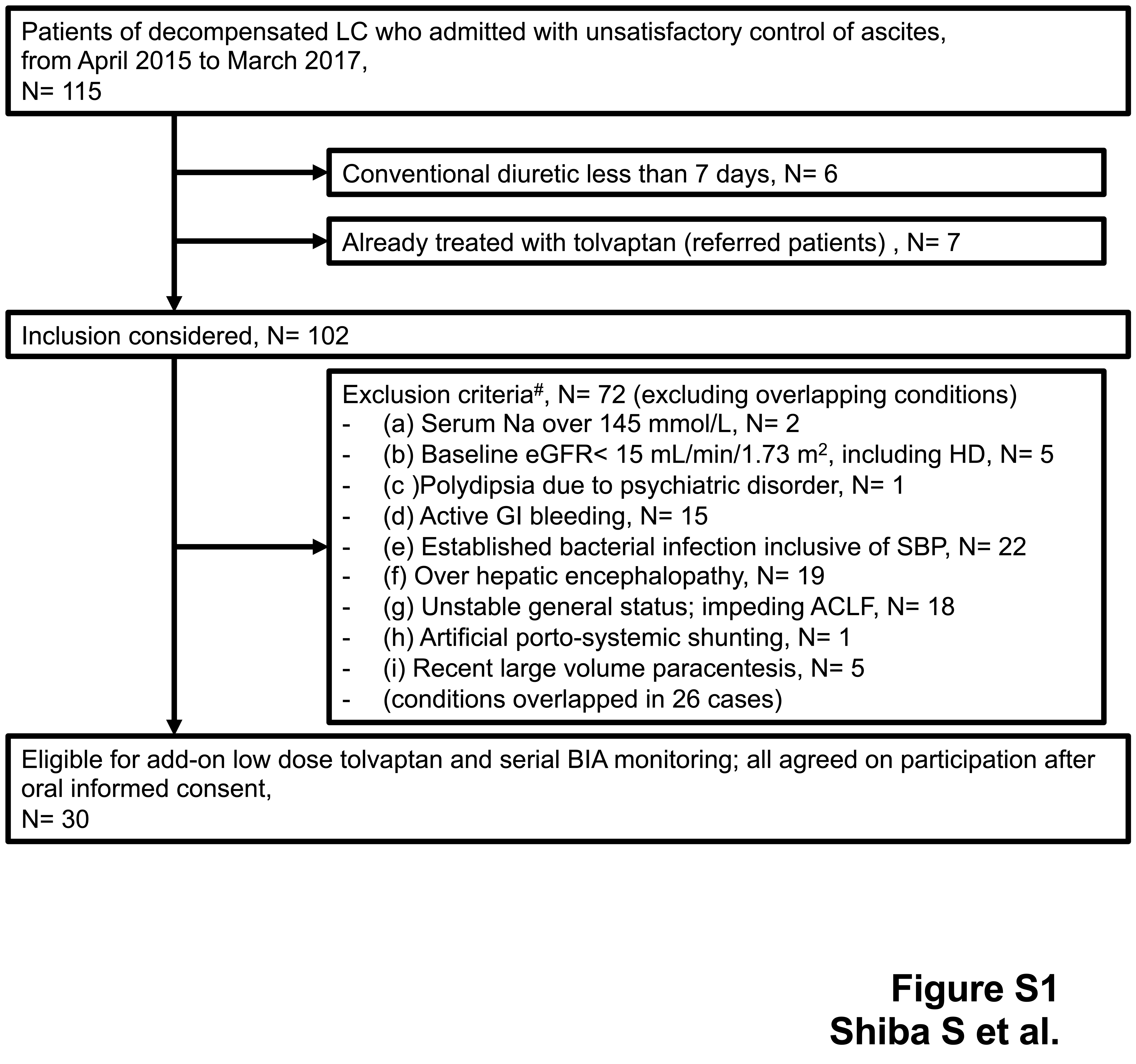
Additional Figures**

**
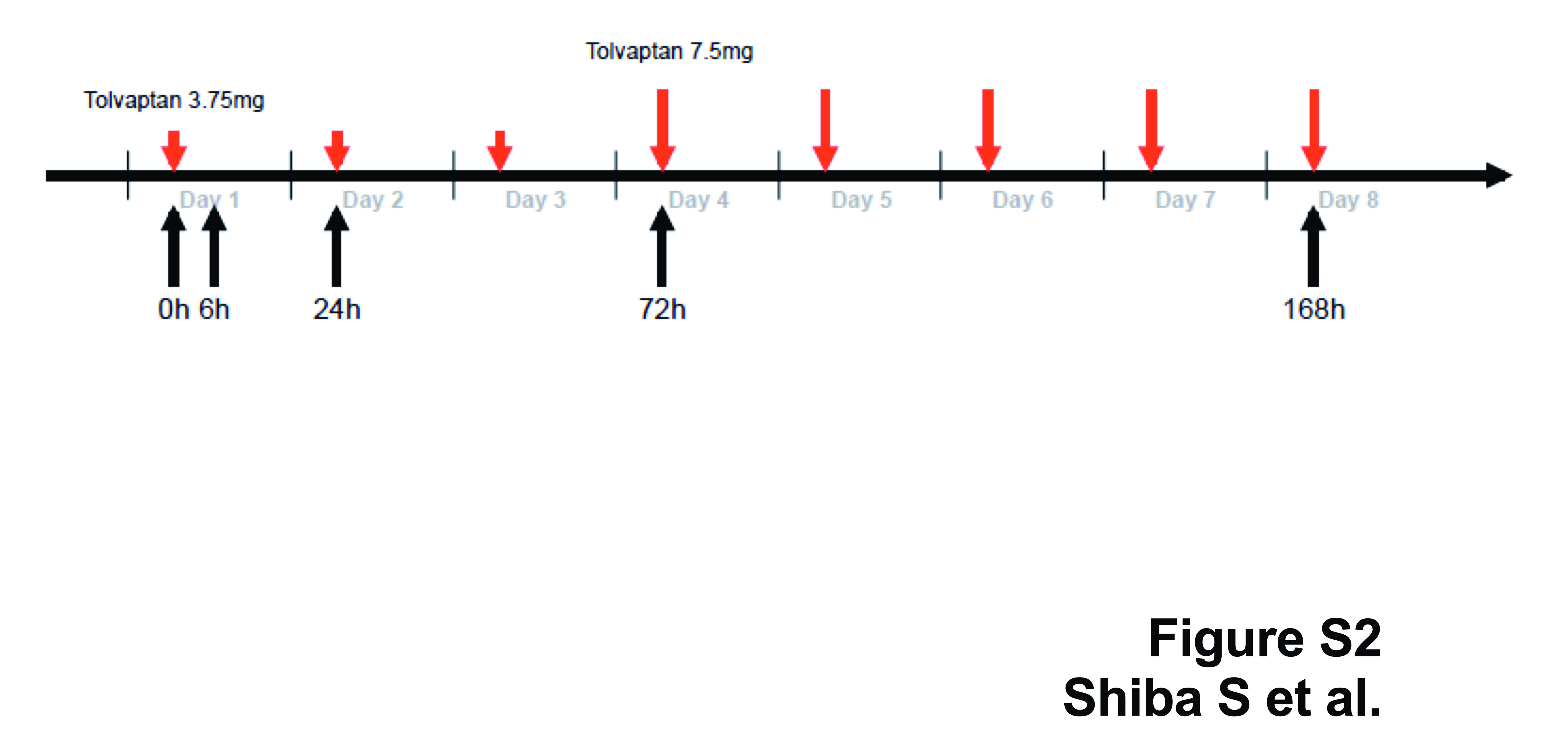
**

**
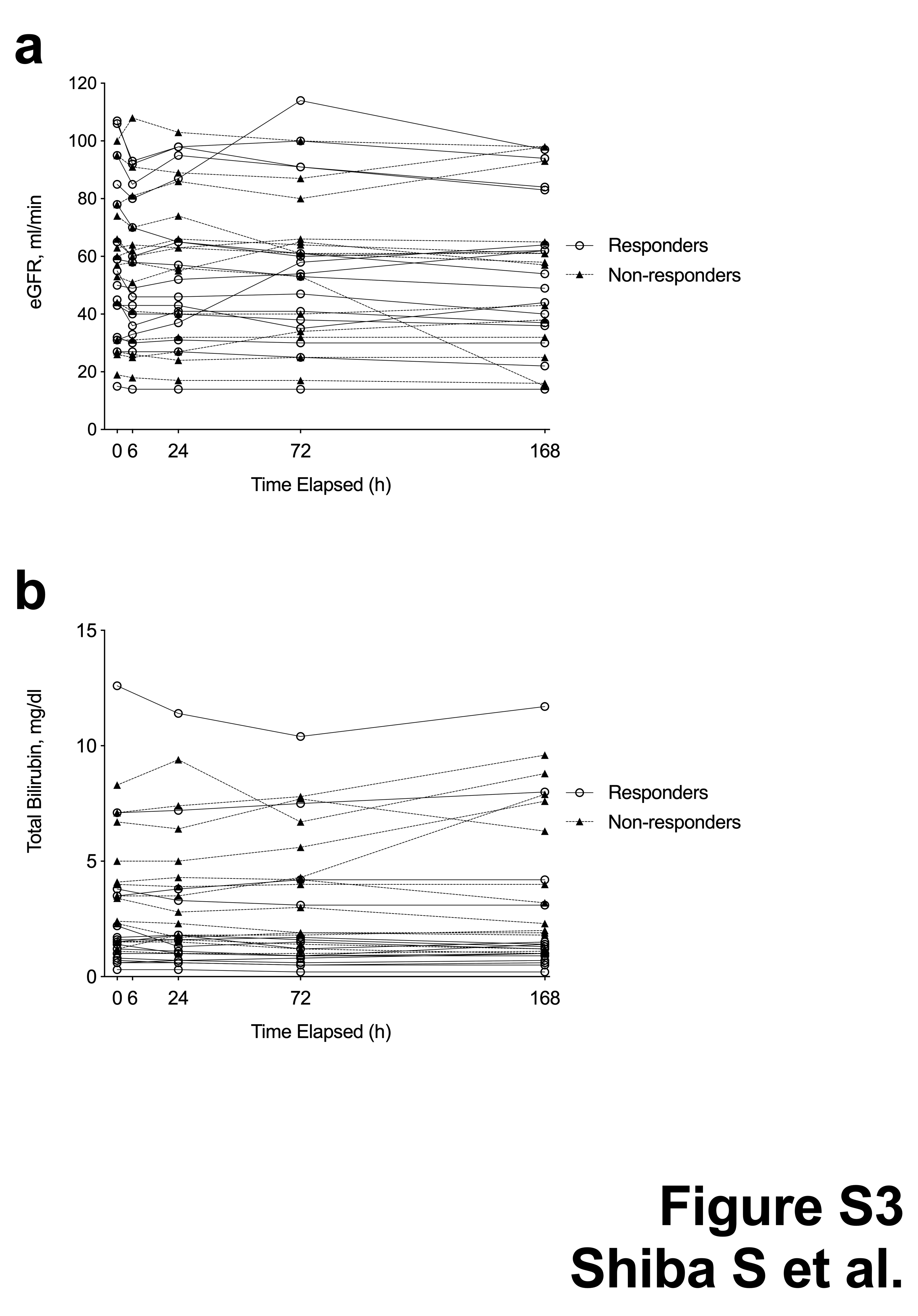
**

**
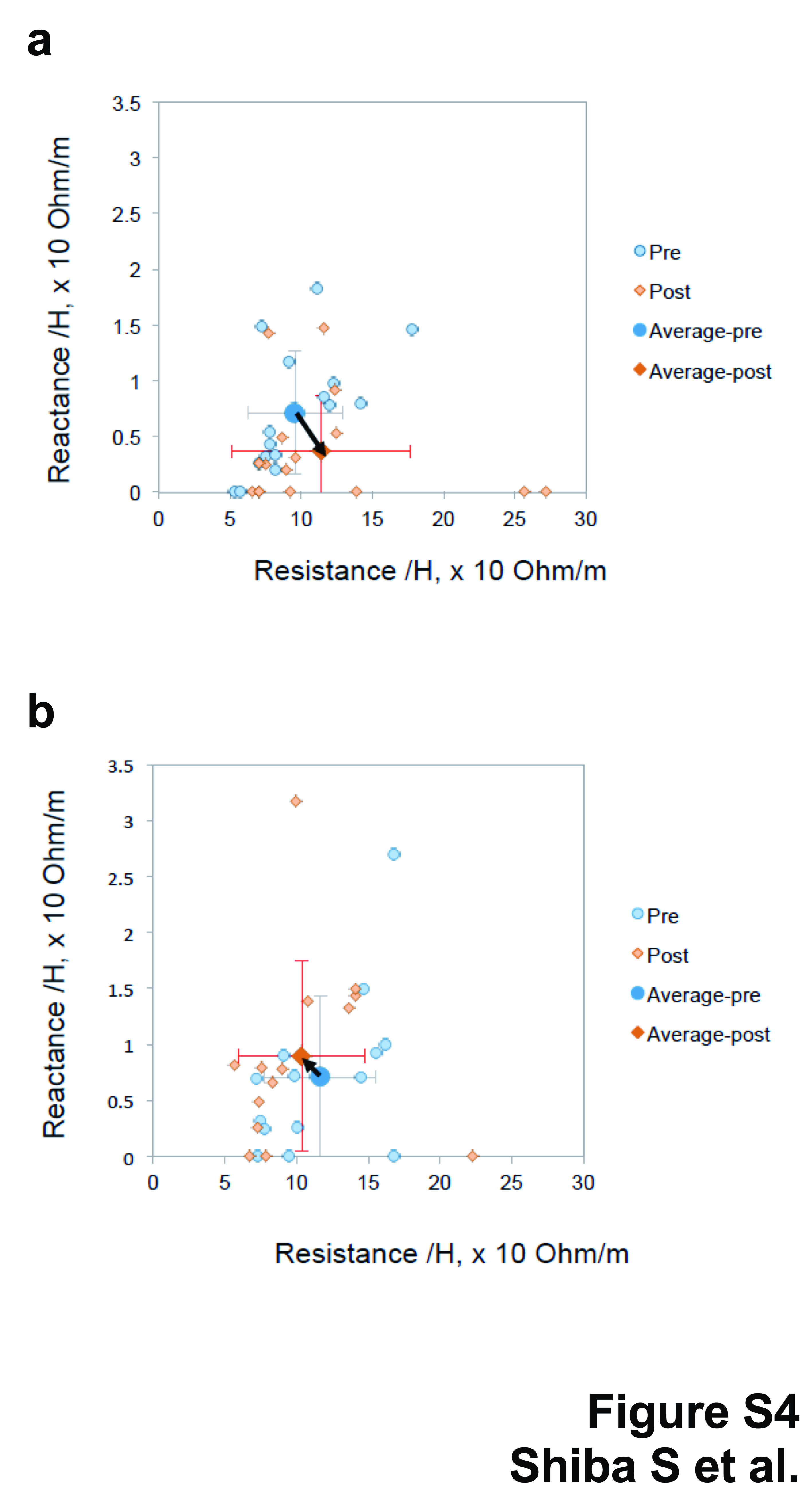
**

**
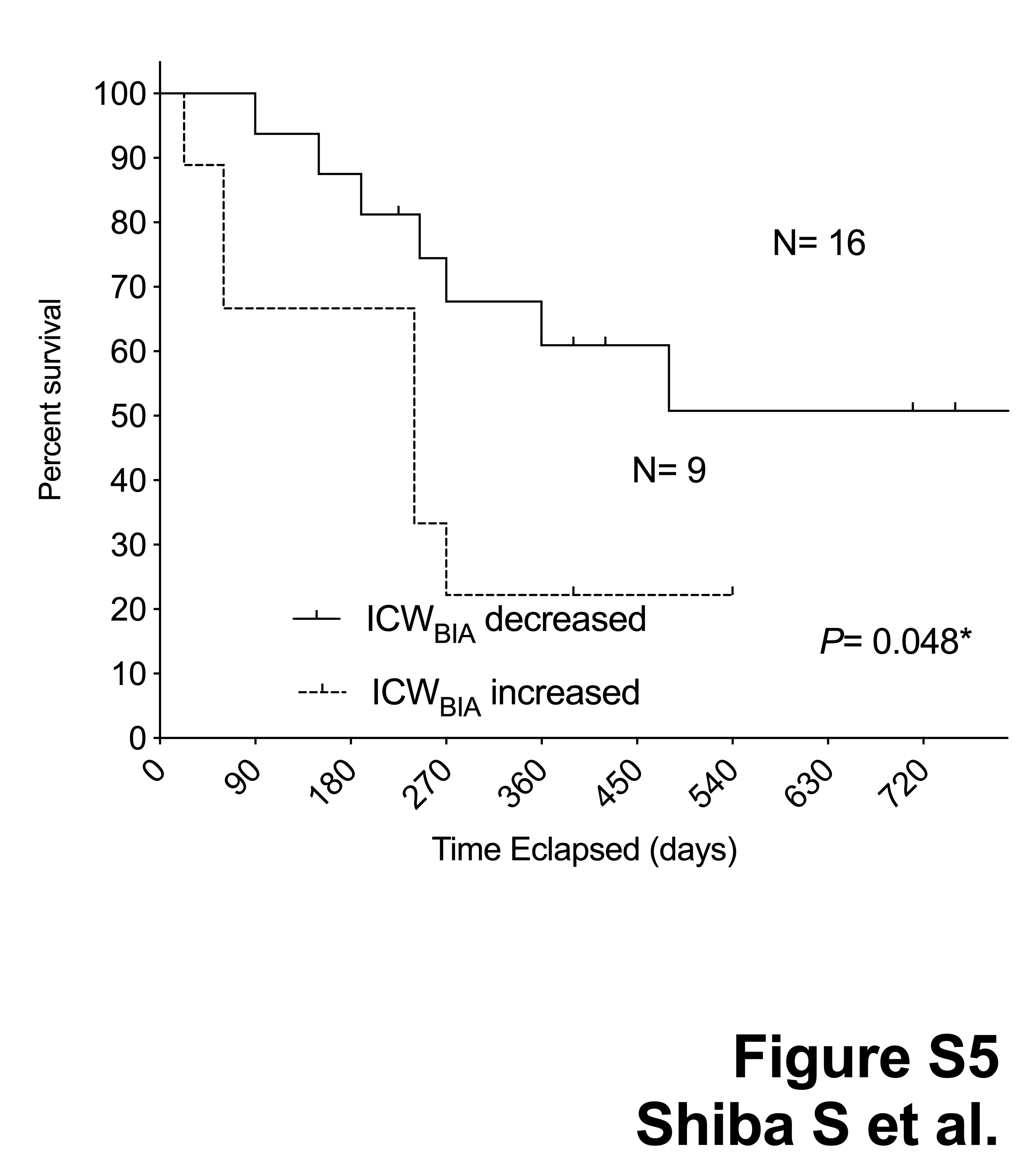
**

**Additional Figure Legends**

**Figure S1**

Inclusion flow of the study subjects.

**Figure S2.**

Study schema for tolvaptan administration and the serial monitoring of body compartments and biochemical studies.

**Figure S3.**

Serial monitoring of estimated glomerular filtration rate and total bilirubin of the study subjects after add-on tolvaptan at low doses.

**Figure S4.**

The resistance-reactance path graphs for responders (panel A) and non-responders (panel B) pre and post add-on tolvaptan at frequency of 50kHz.

**Figure S5.**

Kaplan–Meier analysis for long-term survival as stratified by bioimpedance-defined intracellular water response to add-on tolvaptan in cases without advanced hepatocellular carcinoma.

**Additional Tables**

**Table S1. Pretreatment clinical characteristics regarding hepatocellular carcinoma and portal hypertension of the study subjects**

| **Variables** | **All** | **Responders** | **Non-responders** | ***P-value*** |
| --- | --- | --- | --- | --- |
| **N** | 30 | 16 (53%) | 14 (47%) | - |
| Hepatocellular carcinoma, N/Y | 18/12 | 10/6 | 8/6 | 1.000 |
| None/ BCLC0-A/ BCLC B/ BCLC C | 18/4/3/2/3 | 10/3/2/0/1 | 8/1/1/2/2 | 0.332 |
| Portal vein thrombosis, N/Y | 21/9 | 11/5 | 10/4 | 1.000 |
| Esophageal varices, N/Y | 3/27 | 1/15 | 2/12 | 0.586 |
| β-blocker, N/Y | 24/6 | 12/4 | 12/2 | 0.657 |

Abbreviations: BCLC, Barcelona Clinics of Liver Cancer

**Table S2. Comparison between areas under the receiver operating characteristic curve of clinical parameters for differentiation between responders and non-responders.**

| **Variables** | **Unit** | **AUC** | **95% CI** |  | **Cut-off** | **Sensitivity** | **Specificity** | **PPV** | **NPV** | **Accuracy** | ***P*** | ***P*** |
| --- | --- | --- | --- | --- | --- | --- | --- | --- | --- | --- | --- | --- |
|  |  |  | **Lower** | **Upper** |  | **(%)** | **(%)** | **(%)** | **(%)** | **(%)** |  | **VS ΔICW_BIA_%- 6h** |
| **ΔICW_BIA_%-6h** | % | 0.98 | 0.83 | 1.00 | 0 | 100 | 93 | 94 | 100 | 97 | **<0.0001***** | -- |
| **CPT (score)** | - | 0.69 | 0.49 | 0.86 | 10 | 69 | 64 | 69 | 69 | 67 | 0.06 | **0.006**** |
| **MELD** | - | 0.65 | 0.42 | 0.83 | 16 | 88 | 43 | 64 | 75 | 66 | 0.08 | **0.003**** |
| **T-Bil** | mg/dL | 0.73 | 0.52 | 0.89 | 2.2 | 75 | 71 | 75 | 71 | 73 | 0.24 | **0.023*** |
| **ALT** | IU/L | 0.81 | 0.57 | 0.93 | 22 | 75 | 86 | 86 | 75 | 80 | **0.007**** | **0.098** |
| **AVP** | pg/mL | 0.75 | 0.52 | 0.89 | 1.5 | 63 | 85 | 83 | 65 | 73 | 0.07 | **0.025*** |
| **Aldosterone** | pg/mL | 0.77 | 0.56 | 0.90 | 89 | 44 | 100 | 100 | 59 | 70 | **0.002**** | **0.025*** |

*, *P*< 0.05; **, *P*< 0.01; ***, *P*< 0.0001

Abbreviations: AUC, area under curve; CI, confidence interval; PPV, positive predictive value; NPV, negative predictive value; ICW_BIA_, bioimpedance analysis-defined intracellular water; CPT, Child-Pugh-Turcotte; MELD, model for end-stage liver disease; T-Bil, total bilirubin; ALT, alanine aminotransferase; AVP, arginine vasopressin

**Table S3. Models of multivariate analysis for predicting the rapid decrease of ICW_BIA_**

|  | **Model 1** | | | **Model 2** | | | **Model 3** | | | **Model 4** | | |
| --- | --- | --- | --- | --- | --- | --- | --- | --- | --- | --- | --- | --- |
| **Variables** | **OR** | **95%CI** | ***P*** | **OR** | **95%CI** | ***P*** | **OR** | **95%CI** | ***P*** | **OR** | **95%CI** | ***P*** |
| MELDNa | 0.910 | 0.738-1.090 | 0.305 | 0.872 | 0.718-1.026 | 0.100 | - | - | - | - | - | - |
| Total bilirubin | - | - | - | - | - | - | 0.891 | 0.611-1.327 | 0.536 | 0.823 | 0.584-1.140 | 0.228 |
| ALT | 0.966 | 0.897-1.031 | 0.288 | 0.920 | 0.823-0.995 | **0.035*** | 0.976 | 0.915-1.039 | 0.428 | 0.931 | 0.841-1.003 | 0.063 |
| UN | 0.925 | - | 0.111 | 0.940 | 0.843-1.032 | 0.191 | 0.905 | 0.799-0.001 | **0.030*** | 0.911 | 0.811-0.993 | **0.033*** |
| Aldosterone | 0.995 | 0.987-0.999 | **0.021*** | - | - | - | 0.994 | 0.986-0.999 | **0.013*** | - | - | - |
| AVP | - | - | - | 1.354 | 0.510-4.075 | 0.554 | - | - | - |  |  | 0.417 |

*, *P*< 0.05; **, *P*< 0.01.

MELDNa, model for end-stage liver disease- sodium; T-Bil, total bilirubin; ALT, alanine aminotransferase; AVP, arginine vasopressin

Abbreviations: OR, odds ratio; CI, confidence interval.
